# Stability of targeted metabolite profiles of urine samples under different storage conditions

**DOI:** 10.1007/s11306-016-1137-z

**Published:** 2016-11-28

**Authors:** Markus Rotter, Stefan Brandmaier, Cornelia Prehn, Jonathan Adam, Sylvia Rabstein, Katarzyna Gawrych, Thomas Brüning, Thomas Illig, Heiko Lickert, Jerzy Adamski, Rui Wang-Sattler

**Affiliations:** 1 Research Unit of Molecular Epidemiology, Helmholtz Zentrum München, 85764 München-Neuherberg, Germany; 2 Institute of Epidemiology II, Helmholtz Zentrum München, 85764 München-Neuherberg, Germany; 3 Genome Analysis Center, Institute of Experimental Genetics, Helmholtz Zentrum München, German Research Center for Environmental Health, 85764 München-Neuherberg, Germany; 4 Institute for Prevention and Occupational Medicine of the German Social Accident Insurance, Institute of the Ruhr University Bochum (IPA), 44789 Bochum, Germany; 5 Hannover Unified Biobank, Hannover Medical School, 30625 Hannover, Germany; 6 Institute of Diabetes and Regeneration Research, Helmholtz Zentrum München, 85764 München-Neuherberg, Germany; 7 Institute of Stem Cell Research, Helmholtz Zentrum München, 85764 München-Neuherberg, Germany; 8 German Center for Diabetes Research (DZD), 85764 München-Neuherberg, Germany; 9 Experimental Genetics, Center of Life and Food Sciences Weihenstephan, Technische Universität München, 85354 Freising-Weihenstephan, Germany

**Keywords:** Urine, Storage conditions, Targeted metabolomics, Pre-analytics, Amino acids

## Abstract

**Introduction:**

Few studies have investigated the influence of storage conditions on urine samples and none of them used targeted mass spectrometry (MS).

**Objectives:**

We investigated the stability of metabolite profiles in urine samples under different storage conditions using targeted metabolomics.

**Methods:**

Pooled, fasting urine samples were collected and stored at −80 °C (biobank standard), −20 °C (freezer), 4 °C (fridge), ~9 °C (cool pack), and ~20 °C (room temperature) for 0, 2, 8 and 24 h. Metabolite concentrations were quantified with MS using the AbsoluteIDQ™ p150 assay. We used the Welch-Satterthwaite-test to compare the concentrations of each metabolite. Mixed effects linear regression was used to assess the influence of the interaction of storage time and temperature.

**Results:**

The concentrations of 63 investigated metabolites were stable at −20 and 4 °C for up to 24 h when compared to samples immediately stored at −80 °C. When stored at ~9 °C for 24 h, few amino acids (Arg, Val and Leu/Ile) significantly decreased by 40% in concentration (*P* < 7.9E−04); for an additional three metabolites (Ser, Met, Hexose H1) when stored at ~20 °C reduced up to 60% in concentrations. The concentrations of four more metabolites (Glu, Phe, Pro, and Thr) were found to be significantly influenced when considering the interaction between exposure time and temperature.

**Conclusion:**

Our findings indicate that 78% of quantified metabolites were stable for all examined storage conditions. Particularly, some amino acid concentrations were sensitive to changes after prolonged storage at room temperature. Shipping or storing urine samples on cool packs or at room temperature for more than 8 h and multiple numbers of freeze and thaw cycles should be avoided.

**Electronic supplementary material:**

The online version of this article (doi:10.1007/s11306-016-1137-z) contains supplementary material, which is available to authorized users.

## Introduction

The field of metabolomics has garnered much attention in recent years (Beger et al. 2016; Bouatra et al. 2013). Potential biomarkers for diseases such as type 2 diabetes and metabolite signatures of medication use and lifestyle choices (e.g. smoking) have been identified (Adam et al. 2016; Brandmaier et al. 2015; Wang-Sattler et al. 2012; Xu et al. 2013, 2015). Identified metabolites provide insight into key physiological mechanisms and underlying pathways.

Extensive research on the influence of storage conditions on human plasma and serum metabolite profiles has been conducted (Breier et al. 2014; Anton et al. 2015). A recent study found that serum concentration of 24 out of 127 quantified metabolites significantly changed at room temperature when compared to the values samples stored at −80 °C (Anton et al. 2015). Regarding plasma, the concentration of 44 out of 159 metabolites changed significantly when kept for 24 h at room temperature (Breier et al. 2014).

With respect to its noninvasiveness, metabolomics of urine samples has become a major focus. The collection of such samples can be conducted without supervision of medical experts (Gao 2013). On the other hand, this means that the samples are not necessarily taken under a controlled, well-regulated clinical environment that enhances measurement reproducibility. Therefore, it is pivotal to determine the effects of pre-analytical sample handling, including storage conditions. Previous research on the pre-analytical effects on human urine samples was regarding non-targeted mass spectrometry (MS) or nuclear magnetic resonance (NMR) technology (Barton et al. 2008; Bernini et al. 2011; Budde et al. 2016; Emwas et al. 2015; Gika et al. 2008; Lauridsen et al. 2007; Roux et al. 2015).

In our study, we use a targeted MS approach to investigate the effects of storage conditions, as well as the interaction of storage time and temperature, on urine metabolite concentrations. The urine samples were stored for 2, 8, and 24 h at temperatures ranging from −80 to ~20 °C and exposed to up to three freeze–thaw cycles.

## Materials and methods

### Urine sample preparation under different storage conditions

Urine was collected from six healthy female volunteers between 8:00 and 8:45 am after overnight fasting in sterile disposable containers. To lessen inter-individual differences in urine metabolic profiles, equal parts of urine of each participant were pooled in a sterile 200 ml Erlenmeyer flask and 65 times 1 ml of urine was aliquoted to separate 1.5 ml Eppendorf tubes. Five of these aliquots were immediately frozen at −80 °C and used as baseline reference.

**Fig. 1:**
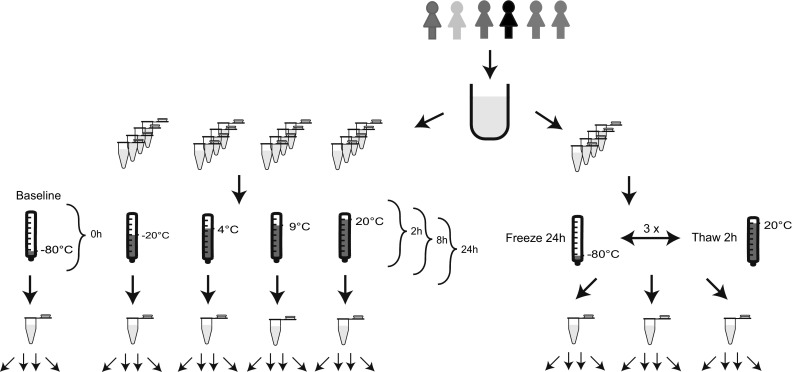
Overview of the study design. Urine from six female volunteers was pooled and aliquoted before being stored at −80, −20, 4, 9, and 20 °C for 0, 2, 8, and 24 h. For freeze and thaw cycles, samples were frozen for 24 h and thawed for 2 h per cycle. Each sample was measured four times

With respect to temperature, we exposed the samples to: (1) room temperature (~20 °C); (2) cool packs (~9 °C); (3) fridge (4 °C) and; (4) freezer (−20 °C). Additionally, with respect to the duration, we stored the samples for 2, 8 and 24 h. This resulted in 12 different conditions. As we prepared four biological replicates, a total of 48 further aliquots were therefore used (Fig. 1).

Additionally, the influence of freezing and thawing urine samples was simulated on the remaining 12 aliquots. Urine was frozen for 24 h at −80 °C and thawed for 2 h at ~20 °C. This cycle was repeated three times (Fig. 1).

### Targeted metabolite quantification

Each sample was measured with the Absolute*IDQ*™ p150 Kit (BIOCRATES Life Sciences AG, Innsbruck, Austria) and FIA-ESI-MS/MS (flow injection-electrospray ionisation-triple quadrupol mass spectrometry). The assay procedures of the Absolute*IDQ*™ p150 Kit have been described in full detail previously (Römisch-Margl et al. 2012). Samples were prepared by a Hamilton Microlab STAR™ robot (Hamilton Bonaduz AG, Bonaduz, Switzerland) and a Ultravap nitrogen evaporator (Porvair Sciences, Leatherhead, UK), beside standard laboratory equipment. Mass spectrometric (MS) analyses were done on an API 4000 LC–MS/MS System (Sciex Deutschland GmbH, Darmstadt, Germany) equipped with a 1200 Series HPLC (Agilent Technologies Deutschland GmbH, Böblingen, Germany) and a HTC PAL auto sampler (CTC Analytics, Zwingen, Switzerland) controlled by the software Analyst 1.6.1. Data evaluation for quantification of metabolite concentrations and quality assessment was performed with the Met*IDQ*™ software package, which is an integral part of the Absolute*IDQ*™ Kit. Metabolite concentrations [µM] were calculated referring to internal standards.

Of 10 µL urine, 162 metabolites were quantified. The baseline reference was measured five times to calculate the coefficients of variance (CV, Table 1). In the course of quality control, we excluded metabolites with a CV higher than 25%. Furthermore, to assure detectability we excluded metabolites with more than 50% of measured values below the limit of detection (three times the median value of water based zero-samples). In total 63 metabolites passed the quality control: free carnitine, 34 acylcarnitines (Cx:y), 13 proteinogenic amino acids, creatinine, hexoses (sum of hexoses), 8 glycerophospholipids (7 phosphatidylcholines (PC) and one lysoPC), and 5 sphingolipids (SM). The abbreviations Cx:y depicts the total number of carbons and double bonds of all chains, respectively (for more details see the list of metabolites in Table S1).

**Table 1:** Significant metabolites identified by the pairwise comparison of baseline concentrations against 24 h at ~20 and ~9 °C

Metabolite	Baseline (0 h)	2 h			8 h			24 h		
	Mean (µM)	Change [%)	*P* value	CV (%)	Change (%)	*P* value	CV (%)	Change (%)	*P* value	CV (%)
~20 °C										
Arg	17.40	1.0	0.82	5.8	−28.3	0.03	17.9	−40.9	**3.1E−4**	10.8
Met	12.65	−7.1	0.26	5.1	−15.3	0.16	18.0	−43.7	**1.3E−4**	3.5
Ser	248.63	−3.7	0.44	4.8	−17.4	0.05	12.3	−36.2	**4.0E−5**	6.6
Val	30.86	−4.9	0.43	5.8	−21.7	0.09	20.3	−59.0	**1.0E−5**	3.5
xLeu	50.39	−6.1	0.25	3.0	−22.1	0.07	19.4	−39.6	**8.8E−5**	3.6
H1	622.83	3.7	0.19	2.2	−3.0	0.67	15.0	−30.3	**9.5E−6**	4.3
~9 °C										
C6:1	0.16	1.9	0.77	9.2	2.2	0.76	10.3	16.2	**8.1E−5**	2.6
Arg	17.40	−0.9	0.91	11.0	6.6	0.29	6.3	−40.2	**7.2E−5**	7.0
Val	30.86	−4.9	0.41	4.4	−2.8	0.69	8.8	−42.5	**4.8E−5**	8.1
xLeu	50.39	−2.9	0.64	7.1	0.2	0.96	8.7	−35.3	**1.1E−4**	4.9

The first column shows metabolites with significant changes in their concentration due to storage conditions when compared to the reference samples that are shown in the second column. The following columns show the percentage of concentration change, the respective *P* value, and coefficient variance (CV) derived with from the four measurements of the samples stored at the specified temperatures/conditions (2, 8, and 24 h at ~20 and ~9 °C). Significant *P* values (*P* < 7.9E−4) are indicated in bold

### Statistical analysis

To account for technical variation due to measurements, each of the 12 replicates and the three samples that underwent freeze and thaw cycles was measured four times. The resulting values were used to calculate the CV under each condition (Table 1).

All metabolite concentrations were log-transformed and standardized (mean = 0 and standard deviation = 1). For each metabolite under each condition, we performed pairwise comparisons by applying a Welch-Satterthwaite separate-variance t test, to assess the differences in metabolite concentration between samples exposed to 2, 8, and 24 h at the respective storage condition with baseline samples (immediately frozen at −80 °C). The same t test was used to pair-wisely compare metabolite concentrations after the freeze and thaw cycles with baseline. To account for multiple testing, Bonferroni correction (*P* < 7.9E−4 = 0.05/63) was applied due to 63 used metabolites.

A mixed effects linear regression model was utilized to assess the influence of time and temperature, as well as their interaction term on metabolite concentrations. Additionally, the variables time and temperature were standardized (mean = 0 and standard deviation = 1). The metabolite concentration was used as the dependent variable, time, temperature and their interaction term as the fixed effect and the repeated measurement as random effect.

The mixed effects linear regression model was used to estimate the impact of the number (0–3) of freeze–thaw cycles on each of the used 63 metabolite concentrations.

Statistical analyses were performed with SAS 9.4 (SAS Institute, Cary NC) using ‘*PROC TTEST*’ for pairwise comparisons of storage conditions and ‘*PROC MIXED*’ for the mixed effect linear regression models on the effect of time, temperature, interaction of time, and temperature and number of freeze and thaw cycles.

## Results

### Amino acids are mostly affected by the storage conditions

**Fig. 2:**
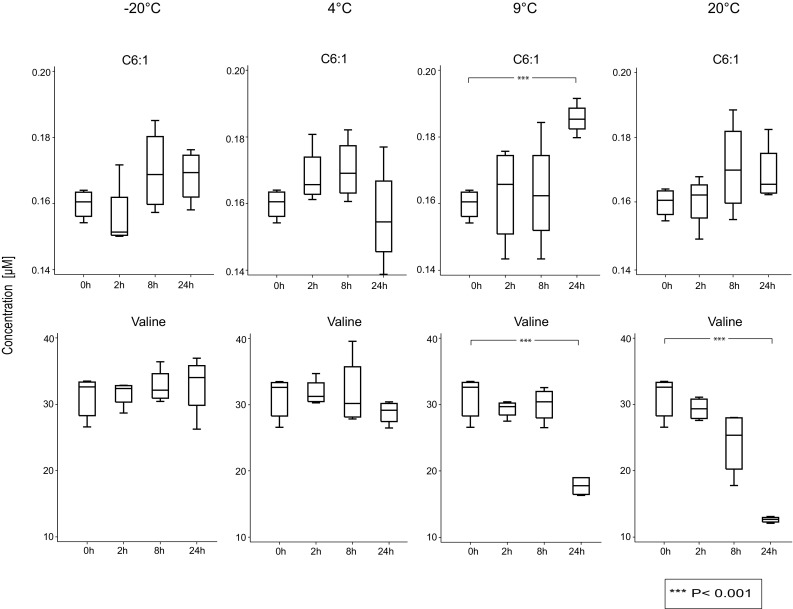
Concentrations of two metabolites over time for various storage conditions. Influence of storage conditions on the concentrations of Valine and C6:1. The concentration of C6:1 at 9 °C increased, whereas the concentration of valine decreased at 20 and 9 °C in urine over the course of 24 h

We observed that the concentrations of about 90% of examined metabolites in the urine samples were not significantly affected by any of the applied storage conditions (for 0, 2, 8, and 24 h at −20, 4, ~9, and ~20 °C, respectively) when compared to samples immediately stored at −80 °C. Only seven out of 63 metabolite concentration measurements were significantly altered. The concentrations of three amino acids were decreased by 35–43% when storing in ~9 °C, and five by up to about 60% when storing the urine samples at room temperature for 24 h (Table 1). No significant changes in the concentration of any metabolite could be observed for the storage at 4 and −20 °C, when compared to baseline. Furthermore, at ~20 °C, no changes in the concentration of the examined 63 metabolites could be observed after 2 and 8 h, but at 24 h Arg, Met, Ser, Val, Leu/Ile, and H1 showed a significant decrease (Table 1). We observed a significant decrease for Arg, Val and Leu/Ile and a significant increase for hexenoylcarnitine (C6:1) at ~9 °C at 24 h (Fig. 2).

### The interaction of temperature and storage time is the most important influence

To further investigate the influence of the combination of time and temperature on the metabolite concentrations, we applied linear mixed effect models. We observed that the concentrations of ten metabolites (Arg, Glu, Met, Phe, Pro, Ser, Thr, Val, Leu/Ile, and H1) showed significant [*P* < 2.6E−4 = 0.05/(63 × 3) to account for three independent variables and 63 metabolites] associations with the interaction term of time and temperature. These ten metabolites included all that were detected with the pairwise comparison of baseline with samples stored at ~20 °C for 24 h (Table 2).

**Table 2:** Metabolites significantly influenced by the interaction of exposure time and temperature

Metabolite	β-estimate (95% CI)	*P* value
Arg	−0.89 (−1.26, −0.52)	**2.2E−5**
Gln	−0.68 (−0.97, −0.40)	**2.5E−5**
Met	−1.00 (−1.31, −0.70)	**1.2E−7**
Phe	−0.71 (−1.03, −0.39)	**7.8E−5**
Pro	−0.83 (−1.20, −0.46)	**5.8E−5**
Ser	−1.01 (−1.26, −0.75)	**9.8E−10**
Thr	−0.69 (−1.01, −0.38)	**7.7E−5**
Val	−1.01 (−1.44, −0.58)	**2.8E−5**
xLeu	−0.95 (−1.28, −0,61)	**1.2E−6**
H1	−1.14 (−1.51, −0.76)	**4.5E−7**

The first column shows metabolites with a significant influence on the interaction of time and temperature. The following columns show β-estimates with respective confidence intervals (95%) and *P* values via a linear mixed effects model. Significant *P* values (*P* < 2.64E−4) are indicated in bold

### Frequent freeze and thaw cycles influences the sample quality

To investigate the effect of freeze and thaw cycles, we conducted pairwise comparisons between samples that underwent up to three cycles with baseline. We did not observe any significantly changed metabolite concentration for one or two freeze and thaw cycles, but two metabolites (H1 and C3) showed significantly increased concentrations for three freeze and thaw cycles, when compared to samples immediately frozen at −80 °C (Table 3). Hexose H1 concentrations increased gradually from baseline (622.83 μM) starting with cycle one (652.28 μM/4.7%) and two (698.83 μM/12.2%) until 748.95 μM/20.2% increase after the third cycle (Table S2). Results depicting the influence of freeze and thaw cycles on all 63 used metabolites are shown in Table S2.

**Table 3:** Metabolites significantly influenced by freeze and thaw cycles

Metabolite	Baseline (0 h)	Cycle 1 (26 h)			Cycle 2 (52 h)			Cycle 3 (78 h)		
	Mean (µM)	Change (%)	*P* value	CV (%)	Change (%)	*P* value	CV (%)	Change (%)	*P* value	CV (%)
C3	1.00	1.3	0.55	2.3	7.7	0.13	7.1	13.8	**7.7E−4**	1.3
H1	622.84	4.7	0.24	5.6	12.2	0.07	8.3	20.2	**3.1E−4**	3.4

The first column shows metabolites that were significantly changed after the third freeze and thaw cycle when compared to the baseline values that are shown in the second column. The following columns show the percentage of concentration change, the respective *P* value, and coefficient variance (CV) derived with from the measurements of the sample after one, two, and three freeze and thaw cycles. Significant *P* values (*P* < 7.9E−4) are indicated in bold

With the linear mixed effect model, we detected a significant association between the number of freeze and thaw cycles and the concentration of four Acylcarnitines (C3, C4, C8:1, C16:1-OH) and Hexose (Table 4).

**Table 4:** Metabolites significantly associated with the number of freeze and thaw cycles

Metabolite	β-estimate (95% CI)	*P* value
C3	0.53 (0.41–1.03)	**2.5E−4**
C4	0.62 (0.36–0.88)	**1.8E−4**
C8:1	0.70 (0.39–1.01)	**2.9E−4**
C16:1-OH	0.72 (0.41–1.03)	**2.5E−4**
H1	0.45 (0.27–0.64)	**1.5E−4**

The first column shows metabolites that were significantly associated with the number of freeze and thaw cycles. The following columns show β-estimates with respective confidence intervals (95%) and *P* values derive with a linear mixed effects model. Significant *P* values (*P* < 7.9E−4) are indicated in bold

## Discussion

We investigated the influence of storage conditions (temperature, time and freeze and thaw cycles) on metabolite profiles in human urine samples using targeted MS and observed that a full day of storing at room temperature or on cool packs significantly altered the concentration of several metabolites, in particular amino acids. This finding was confirmed by investigating the interaction between exposure time and temperature. Furthermore, we observed that more than two freeze and thaw cycles affected the metabolite concentrations in the urine samples. However, about 78% of quantified metabolites in urine samples from overnight fasting females were not influenced by the examined storage conditions, when considering a stringent Bonferroni corrected level of significance.

Although other studies investigated the impact of storage conditions on metabolite profiles in urine as well, they were using either non-targeted MS or NMR technology (Table 5). Moreover, none of these studies investigated the interaction between storage time and temperature, which previously was only subject to research on the storage of serum samples (Anton et al. 2015).

**Table 5:** Previous studies on pre-analytical sample handling

Author	Technique	Approach	Tissue	Temperature	Time	Interaction	Freeze/thaw cycles
Lauridsen et al. (2007)	NMR	Non-targeted	Urine	−80, −25, and 4 °C	0, 1, 2, 3, 4, 6, 10, 14, and 26 weeks	–	–
Barton et al. (2008)	NMR	Non-targeted	Urine, serum	−80 and 4 °C	0, 24, and 36 h	–	–
Bernini et al. (2011)	NMR	Non-targeted	Urine, serum plasma	−80, 4 °C, and RT	0, 2, 4, 6, 24 h, and 1 week	–	–
Roux et al. (2015)	NMR, MS	Non-targeted	Urine	−80, 4 °C, and RT (19–26 °C)	0–72 h (every 4 or 12 h)	–	–
Budde et al. (2016)	NMR	Non-targeted	Urine	−80, 4, 10 °C, and RT (25 °C)	0, 1, 2, 8, 10, 12, 24, 28, 72 h, and 1 month	–	–
Gika et al. (2008)	MS	Non-targeted	Urine	−80, −20, and −4 °C,	1 week, 1 month, 3 months, and 6 months	–	X (−20 °C)
Breier et al. (2014)	MS	Targeted	Serum, plasma	−80, ~4 °C, and RT (21 °C)	0, 3, 6, and 24 h	–	X (−20 °C)
Anton et al. (2015)	MS	Targeted	Serum	−80 °C, dry ice, wet ice, and RT (22–24 °C)	0, 12, 24, and 36 h	X	X (−80 °C)
Rotter et al.	MS	Targeted	Urine	−80, −20, 4, ~9 °C, and RT (~20 °C)	0, 2, 8, and 24 h	X	X (−80 °C)

The first three columns indicate the underlying study and the technical approach that was applied. The following columns depict the examined tissue and the pre-processing conditions (temperature, time, and their interaction), the samples were exposed to. The final column indicates if freeze and thaw cycles were subject to the respective study

*RT* room temperature, *NMR* nuclear magnetic resonance, *MS* mass spectrometry

Our finding of decreased concentrations of Hexose in urine at a prolonged exposure to room temperature is consistent with previous studies in plasma and most likely results from active glycolysis enzymes in urine (Breier et al. 2014; Bruns and Knowler 2009; Grötsch et al. 1985). Furthermore, our observation of decreased concentrations of arginine and methionine in urine is consistent with previous observations in plasma at room temperature (Breier et al. 2014).

The decreased concentrations of the branched chain amino acids (BCAA, i.e. valine, leucine and isoleucine) might be explained by the catabolic activity of a multiple enzyme complex, in particular the branched-chain α-keto acid dehydrogenase (BCKDC). BCKDC converts all three amino acid by: (1) transamination; (2) oxidative decarboxylation; and (3) dehydrogenation (Tanaka and Rosenberg 1983). Indo et al. report BCKDC to be associated with the mitochondrial inner membrane (Indo et al. 1987). Prolonged exposure of urine to room temperature might lead to a degradation of cells and a release of BCKDC. The reduction in the concentration of these BCAA, when stored on cool packs is less profound, when compared to room temperature. The comparably smaller effect is most likely due to the reduced temperature (Gillim et al. 1983).

Contradictory findings were reported for blood: the concentrations of leucine, isoleucine and serine in plasma were found to be increased when stored for 24 h at room temperature and for leucine when stored for 24 h on cool packs (Breier et al. 2014). However this is expected, when comparing different matrices, such as blood and urine. Urine at prolonged room temperature is prone to getting more acidic, whereas blood is buffered (Alguacil et al. 2007). Additionally, the number of cells is not comparable between the two matrices, which also accounts for the protein concentration and numerous other factors, such as bacterial growth. To avoid such interferences by reducing the number of bacteria and host cells, as well as large particles, previous studies suggested to pre-centrifuge (1000–3000 RCF for 5 min at 4 °C) and filter urine samples (using a 0.20 µm filter) before conducting the metabolite profiling (Bernini et al. 2011; Emwas et al. 2015). However, certain circumstances like the non-availability of filters and centrifuges and time consuming operation during real life studies make it difficult to use said filters directly after donation.

We observed up to 60% reduction of BCAA (isoleucine and valine) when urine samples are stored on cool packs or at room temperature for 24 h. These BCAA are in particular essential for the growth of certain microorganisms, such as *lactobacillus brevis* and *lactobacillus plantarum* (Katina 2005). *L. brevis* can be found in intestines, colon and vagina (Makarova et al. 2006). The interplay of time and temperature may have led to a consumption and consequent concentration reduction of the respective amino acids in urine samples.

The detected increase in Hexose (mainly glucose) concentration after multiple freeze and thaw cycles might be due to the reported degeneration of sucrose. The enzyme invertase, also called sucrase, catalyzes the hydrolysis of sucrose to glucose and fructose (Huang et al. 1999; Zhang et al. 2016). Invertase is found in the potential urine contaminant yeast (Fisher et al. 1995). Repeated freezing and thawing might have damaged these cells, led to a diffusion of invertase in urine and consequently the hydrolysis of sucrose. Additionally, the acid-catalyzed hydrolysis of sucrose was reported to be enhanced by freezing (Lund et al. 1969). Both mechanisms are likely to explain the gradual increase of glucose concentrations (up to 20%) after the freeze and thaw cycles.

However, the observation of an opposite trend for the glucose concentration after freeze and thaw cycles, when compared to the storing at room temperature for 24 h needs to be further investigated.

In general, using targeted MS approach we observed changes in six metabolites (C3, C6:1, Arg, Val, Leu/Iso (xLeu), H1) that were not reported in other studies that applied similar storage conditions (e.g. 24 h at 10 °C, up to nine freeze and thaw cycles), but different measurement techniques (NMR, non-targeted MS) (Budde et al. 2016; Gika et al. 2008). Other observed alterations in the metabolite profiles were derived under conditions that were not part of our study (e.g. 72 h or 12 weeks at 4 °C) (Lauridsen et al. 2007; Roux et al. 2015). Due to the comparably small number of measurements, our study is limited in statistical power. Additionally, the Absolute*IDQ*™ p150 kit was originally developed for blood samples, and not focusing on urine. This is reflected by the number of only 63 metabolites that passed the quality control. In order to make the kit more applicable to urine, creatinine was included by the manufacturer into the metabolite panel, to enable researchers to account and normalize for different urine excretion rates. Furthermore, the measured values were not derived from biological replicates, but from repeated measurements of the same samples. However, by this procedure, potential analytical variations could be identified and accounted for. Furthermore, by using pooled samples, we do not have to account for different excretion rates and differences in interpersonal metabolite profiles. This supports the direct comparability of measured effects.

## Conclusions

The findings from our study suggested to avoid shipping urine samples on cool packs or at room temperature for durations of more than 8 h, and we have provided insight on improved planning and sample maintenance in the field. We strongly recommend storage temperatures of at least −20 °C and to minimize the number of freeze and thaw cycles to ensure integrity of urine samples used for metabolomics studies.

## Electronic supplementary material

Below is the link to the electronic supplementary material.

Supplementary material 1 (DOC 350 kb)
