# Supplementary material for: Stability of targeted metabolite profiles of urine samples under different storage conditions
Source: Metabolomics. 2016 Nov 28;13(1):4. doi: 10.1007/s11306-016-1137-z (PMC5126183; doi:10.1007/s11306-016-1137-z)
Supplement: Supplementary file 1 — Supplementary material 1 (DOC 350 kb) [file 11306_2016_1137_MOESM1_ESM.doc]

**Table S1. Overview of 163 measured metabolites**

The first and second column depicts the metabolite abbreviation and biochemical name. The third column shows the percentage of values below the limit of detection (LOD, based on concentration of water based zero samples). The following column illustrates the coefficient of variance (CV, based on baseline concentrations). The column labeled Application indicates if the respective metabolite was used for further analysis. A metabolite was considered for analysis if the 50% (or less) of measurements were below LOD and CV was 25% or lower. The last two columns indicate the mean concentrations of baseline values and water based zero samples.

| Metabolite | Biochemical name | % measure-ments below LOD | % CV | Application | Mean concentration baseline | Mean concentration water based zero samples |
| --- | --- | --- | --- | --- | --- | --- |
| C0 | Carnitine | 0.0 | 3.3 | Used | 58.36824 | 1.07099 |
| C2 | Acetylcarnitine | 0.0 | 6.3 | Used | 0.317165 | 0.04 |
| C3 | Propionylcarnitine | 0.0 | 3.8 | Used | 1.043836 | 0.02177 |
| C3:OH | Hydroxypropionylcarnitine | 0.0 | 8.7 | Used | 0.871927 | 0.01029 |
| C3:1 | Propenonylcarnitine | 3.7 | 16.4 | Used | 0.273509 | 0.03647 |
| C4 | Butyrylcarnitine | 0.0 | 4.0 | Used | 0.122693 | 0.11138 |
| (C3-DC)C4-OH | Hydroxybutyrylcarnitine | 0.0 | 4.0 | Used | 0.284414 | 0.08902 |
| C4:1 | Butenylcarnitine | 0.0 | 4.7 | Used | 0.033485 | 0.00854 |
| C5 | Valerylcarnitine | 0.0 | 6.4 | Used | 0.025788 | 0.0047 |
| C5-DC (C6-OH) | Glutarylcarnitine (Hydroxyhexanoylcarnitine) | 0.0 | 5.5 | Used | 0.024804 | 0.00409 |
| C5-M-DC | Methylglutarylcarnitine | 0.0 | 6.4 | Used | 0.026918 | 0.00285 |
| C5-OH (C3-DC-M) | Hydroxyvalerylcarnitine (Methylmalonylcarnitine) | 0.0 | 4.0 | Used | 0.025383 | 0.00311 |
| C5:1 | Tiglylcarnitine | 0.0 | 4.8 | Used | 0.025108 | 0.00476 |
| C5:1-DC | Glutaconylcarnitine | 0.0 | 8.4 | Used | 0.01262 | 0.00265 |
| C6 (C4:1-DC) | Hexanoylcarnitine (Fumarylcarnitine) | 0.0 | 7.6 | Used | 0.051747 | 0.06556 |
| C6:1 | Hexenoylcarnitine | 0.0 | 2.7 | Used | 0.008343 | 0.00298 |
| C7-DC | Pimelylcarnitine | 0.0 | 7.3 | Used | 0.012213 | 0.00224 |
| C8 | Octanoylcarnitine | 0.0 | 8.3 | Used | 0.015714 | 0.00813 |
| C8:1 | Octenoylcarnitine | 0.0 | 6.8 | Used | 0.009326 | 0.0037 |
| C9 | Nonaylcarnitine | 0.0 | 5.6 | Used | 0.007397 | 0.00448 |
| C10 | Decanoylcarnitine | 0.0 | 4.3 | Used | 0.011173 | 0.00767 |
| C10:1 | Decenoylcarnitine | 0.0 | 3.0 | Used | 0.00676 | 0.00141 |
| C10:2 | Decadienylcarnitine | 0.0 | 3.7 | Used | 13.80881 | 0.02079 |
| C12 | Dodecanoylcarnitine | 0.0 | 6.4 | Used | 1.003427 | 0.00968 |
| C12:DC | Dodecanedioylcarnitine | 100.0 | 6.3 | Excluded | 1.197275 | 0.02162 |
| C12:1 | Dodecenoylcarnitine | 0.0 | 6.0 | Used | 0.095798 | 0.01204 |
| C14 | Tetradecanoylcarnitine | 0.0 | 7.4 | Used | 0.027469 | 0.00791 |
| C14:1 | Tetradecenoylcarnitine | 0.0 | 7.9 | Used | 7.085962 | 0.01014 |
| C14:1-OH | Hydroxytetradecenoylcarnitine | 0.0 | 9.5 | Used | 0.184061 | 0.00708 |
| C14:2 | Tetradecadienylcarnitine | 0.0 | 10.6 | Used | 0.186356 | 0.00847 |
| C14:2-OH | Hydroxytetradecadienylcarnitine | 0.0 | 8.5 | Used | 2.392026 | 0.02051 |
| C16 | Hexadecanoylcarnitine | 0.0 | 8.4 | Used | 0.785741 | 0.00627 |
| C16:OH | Hydroxyhexadecanoylcarnitine | 0.0 | 10.1 | Used | 0.652544 | 0.02291 |
| C16:1 | Hexadecenoylcarnitine | 100.0 | 5.3 | Excluded | 1.179905 | 0.03139 |
| C16:1-OH | Hydroxyhexadecenoylcarnitine | 4.9 | 13.9 | Used | 0.40108 | 0.00639 |
| C16:2 | Hexadecadienylcarnitine | 0.0 | 11.2 | Used | 0.559509 | 0.01956 |
| C16:2-OH | Hydroxyhexadecadienylcarnitine | 100.0 | 3.7 | Excluded | 0.15967 | 0.01354 |
| C18 | Octadecanoylcarnitine | 98.8 | 23.1 | Excluded | 0.475602 | 0.00772 |
| C18:1 | Octadecenoylcarnitine | 100.0 | 17.0 | Excluded | 0.555841 | 0.04522 |
| C18:1-OH | Hydroxyoctadecenoylcarnitine | 100.0 | 8.6 | Excluded | 2.449443 | 0.00525 |
| C18:2 | Octadecadienylcarnitine | 0.0 | 10.3 | Used | 2.432516 | 0.00617 |
| Arg | Arginine | 0.0 | 10.6 | Used | 17.40325 | 0.66233 |
| Gln | Glutamine | 0.0 | 6.2 | Used | 350.2045 | 0.64671 |
| Gly | Glycine | 0.0 | 7.8 | Used | 1631.4 | 1.09192 |
| His | Histidine | 0.0 | 7.4 | Used | 332.4206 | 0.1937 |
| Met | Methionine | 0.0 | 11.1 | Used | 12.65441 | 0.52398 |
| Phe | Phenylalanine | 0.0 | 8.3 | Used | 26.27359 | 0.06309 |
| Pro | Proline | 0.0 | 7.2 | Used | 21.92753 | 0.36625 |
| Ser | Serine | 0.0 | 8.1 | Used | 248.628 | 1.14356 |
| Thr | Threonine | 0.0 | 9.6 | Used | 82.35513 | 0.61713 |
| Trp | Tryptophan | 0.0 | 8.8 | Used | 65.92132 | 3.65066 |
| Tyr | Tyrosine | 0.0 | 8.6 | Used | 72.30163 | 0.72567 |
| Val | Valine | 0.0 | 10.3 | Used | 30.85577 | 0.51273 |
| xLeu | Leucine/Isoleucine | 0.0 | 9.5 | Used | 50.39062 | 0.42091 |
| Creatinin | Creatinin | 0.0 | 5.5 | Used | 9405.45 | 7.79205 |
| PC aa C24:0 | Phosphatidylcholine diacyl C24:0 | 100.0 | 18.9 | Excluded | 2.154788 | 1.89108 |
| PC aa C26:0 | Phosphatidylcholine diacyl C26:0 | 100.0 | 3.8 | Excluded | 0.055007 | 0.09234 |
| PC aa C28:1 | Phosphatidylcholine diacyl C28:1 | 0.0 | 7.1 | Used | 0.038754 | 0.02055 |
| PC aa C30:0 | Phosphatidylcholine diacyl C30:0 | 100.0 | 5.2 | Excluded | 0.026802 | 0.00827 |
| PC aa C30:2 | Phosphatidylcholine diacyl C30:2 | 59.3 | 67 | Excluded | 0.088769 | 0.07181 |
| PC aa C32:0 | Phosphatidylcholine diacyl C32:0 | 22.2 | 18.2 | Used | 0.027462 | 0.00765 |
| PC aa C32:1 | Phosphatidylcholine diacyl C32:1 | 60.5 | 30.8 | Excluded | 0.033038 | 0.00884 |
| PC aa C32:2 | Phosphatidylcholine diacyl C32:2 | 27.2 | 89.3 | Excluded | 0.082059 | 0.05234 |
| PC aa C32:3 | Phosphatidylcholine diacyl C32:3 | 35.8 | 52.7 | Excluded | 0.010392 | 0.00479 |
| PC aa C34:1 | Phosphatidylcholine diacyl C34:1 | 0.0 | 22.4 | Used | 0.300634 | 0.27901 |
| PC aa C34:2 | Phosphatidylcholine diacyl C34:2 | 0.0 | 50.8 | Excluded | 0.010508 | 0.00552 |
| PC aa C34:3 | Phosphatidylcholine diacyl C34:3 | 100.0 | 43.4 | Excluded | 0.011559 | 0.00458 |
| PC aa C34:4 | Phosphatidylcholine diacyl C34:4 | 24.7 | 19.4 | Used | 0.061801 | 0.0516 |
| PC aa C36:0 | Phosphatidylcholine diacyl C36:0 | 100.0 | 5.8 | Excluded | 0.027651 | 0.05243 |
| PC aa C36:1 | Phosphatidylcholine diacyl C36:1 | 0.0 | 19.8 | Used | 0.073185 | 0.00553 |
| PC aa C36:2 | Phosphatidylcholine diacyl C36:2 | 0.0 | 33.4 | Excluded | 0.018299 | 0.01462 |
| PC aa C36:3 | Phosphatidylcholine diacyl C36:3 | 0.0 | 54.4 | Excluded | 0.382213 | 0.40453 |
| PC aa C36:4 | Phosphatidylcholine diacyl C36:4 | 7.4 | 47.9 | Excluded | 0.079977 | 0.00729 |
| PC aa C36:5 | Phosphatidylcholine diacyl C36:5 | 61.7 | 33 | Excluded | 0.052186 | 0.04585 |
| PC aa C36:6 | Phosphatidylcholine diacyl C36:6 | 96.3 | 70.6 | Excluded | 0.002066 | 0.00073 |
| PC aa C38:0 | Phosphatidylcholine diacyl C38:0 | 100.0 | 16.2 | Excluded | 0.017631 | 0.00536 |
| PC aa C38:1 | Phosphatidylcholine diacyl C38:1 | 74.1 | 125.3 | Excluded | 0.007773 | 0.00277 |
| PC aa C38:3 | Phosphatidylcholine diacyl C38:3 | 0.0 | 34.8 | Excluded | 0.002046 | 0.00063 |
| PC aa C38:4 | Phosphatidylcholine diacyl C38:4 | 2.5 | 72.5 | Excluded | 0.001772 | 0.0005 |
| PC aa C38:5 | Phosphatidylcholine diacyl C38:5 | 79.0 | 58.3 | Excluded | 0.084161 | 0.00757 |
| PC aa C38:6 | Phosphatidylcholine diacyl C38:6 | 3.7 | 35.4 | Excluded | 0.053297 | 0.00402 |
| PC aa C40:1 | Phosphatidylcholine diacyl C40:1 | 100.0 | 15.0 | Excluded | 0.004708 | 0.00383 |
| PC aa C40:2 | Phosphatidylcholine diacyl C40:2 | 100.0 | 13.4 | Excluded | 0.008329 | 0.00222 |
| PC aa C40:3 | Phosphatidylcholine diacyl C40:3 | 82.7 | 44.1 | Excluded | 0.048148 | 0.04568 |
| PC aa C40:4 | Phosphatidylcholine diacyl C40:4 | 100.0 | 33.2 | Excluded | 0.042108 | 0.00225 |
| PC aa C40:5 | Phosphatidylcholine diacyl C40:5 | 100.0 | . | Excluded | 0.044944 | 0.00393 |
| PC aa C40:6 | Phosphatidylcholine diacyl C40:6 | 100.0 | 6.6 | Excluded | 0.020697 | 0.00211 |
| PC aa C42:0 | Phosphatidylcholine diacyl C42:0 | 100.0 | 17.2 | Excluded | 0.02347 | 0.00399 |
| PC aa C42:1 | Phosphatidylcholine diacyl C42:1 | 100.0 | 20.3 | Excluded | 0.002963 | 0.00117 |
| PC aa C42:2 | Phosphatidylcholine diacyl C42:2 | 100.0 | 4.9 | Excluded | 0.003432 | 0.00213 |
| PC aa C42:4 | Phosphatidylcholine diacyl C42:4 | 100.0 | 41.9 | Excluded | 0.012551 | 0.00981 |
| PC aa C42:5 | Phosphatidylcholine diacyl C42:5 | 88.9 | 66.5 | Excluded | 0.002435 | 0.00256 |
| PC aa C42:6 | Phosphatidylcholine diacyl C42:6 | 100.0 | 6.3 | Excluded | 0.013188 | 0.00228 |
| PC ae C30:0 | Phosphatidylcholine acyl-alkyl C30:0 | 100.0 | 10.2 | Excluded | 0.014346 | 0.00248 |
| PC ae C30:1 | Phosphatidylcholine acyl-alkyl C30:1 | 29.6 | 223.6 | Excluded | 0.007667 | 0.00216 |
| PC ae C30:2 | Phosphatidylcholine acyl-alkyl C30:2 | 100.0 | 4.1 | Excluded | 0.012063 | 0.00195 |
| PC ae C32:1 | Phosphatidylcholine acyl-alkyl C32:1 | 19.8 | 33.5 | Excluded | 0.127762 | 0.12867 |
| PC ae C32:2 | Phosphatidylcholine acyl-alkyl C32:2 | 100.0 | 29.5 | Excluded | 0.009948 | 0.00719 |
| PC ae C34:0 | Phosphatidylcholine acyl-alkyl C34:0 | 98.8 | 31.1 | Excluded | 0.001914 | 0.00103 |
| PC ae C34:1 | Phosphatidylcholine acyl-alkyl C34:1 | 0.0 | 16.7 | Used | 0.00506 | 0.00451 |
| PC ae C34:2 | Phosphatidylcholine acyl-alkyl C34:2 | 9.9 | 30.8 | Excluded | 0 | 0 |
| PC ae C34:3 | Phosphatidylcholine acyl-alkyl C34:3 | 71.6 | 55.4 | Excluded | 0.074016 | 0.07421 |
| PC ae C36:0 | Phosphatidylcholine acyl-alkyl C36:0 | 100.0 | 16.3 | Excluded | 0.021463 | 0.03976 |
| PC ae C36:1 | Phosphatidylcholine acyl-alkyl C36:1 | 100.0 | 14.6 | Excluded | 0.007541 | 0.00486 |
| PC ae C36:2 | Phosphatidylcholine acyl-alkyl C36:2 | 100.0 | 19.8 | Excluded | 0.036747 | 0.03251 |
| PC ae C36:3 | Phosphatidylcholine acyl-alkyl C36:3 | 23.5 | 43.8 | Excluded | 0.00539 | 0.00458 |
| PC ae C36:4 | Phosphatidylcholine acyl-alkyl C36:4 | 69.1 | 11.9 | Excluded | 0.007073 | 0.00346 |
| PC ae C36:5 | Phosphatidylcholine acyl-alkyl C36:5 | 100.0 | 62.4 | Excluded | 0.080159 | 0.09246 |
| PC ae C38:0 | Phosphatidylcholine acyl-alkyl C38:0 | 100.0 | 16.4 | Excluded | 0.039025 | 0.03914 |
| PC ae C38:1 | Phosphatidylcholine acyl-alkyl C38:1 | 87.7 | 101.3 | Excluded | 0.000579 | 0 |
| PC ae C38:2 | Phosphatidylcholine acyl-alkyl C38:2 | 59.3 | 29.6 | Excluded | 0.112452 | 0.11335 |
| PC ae C38:3 | Phosphatidylcholine acyl-alkyl C38:3 | 8.6 | 28.2 | Excluded | 0.005646 | 0.0012 |
| PC ae C38:4 | Phosphatidylcholine acyl-alkyl C38:4 | 54.3 | 67.9 | Excluded | 0.0072 | 0.00776 |
| PC ae C38:5 | Phosphatidylcholine acyl-alkyl C38:5 | 100.0 | 9.9 | Excluded | 0.00662 | 0.00429 |
| PC ae C38:6 | Phosphatidylcholine acyl-alkyl C38:6 | 0.0 | 11.5 | Used | 0.013616 | 0.00342 |
| PC ae C40:1 | Phosphatidylcholine acyl-alkyl C40:1 | 100.0 | 23.6 | Excluded | 0.007925 | 0.0014 |
| PC ae C40:2 | Phosphatidylcholine acyl-alkyl C40:2 | 14.8 | 26.2 | Excluded | 0.003016 | 0.00154 |
| PC ae C40:3 | Phosphatidylcholine acyl-alkyl C40:3 | 4.9 | 54.5 | Excluded | 0.03413 | 0.10941 |
| PC ae C40:4 | Phosphatidylcholine acyl-alkyl C40:4 | 100.0 | 12.2 | Excluded | 0.023548 | 0.01431 |
| PC ae C40:5 | Phosphatidylcholine acyl-alkyl C40:5 | 96.3 | 43.4 | Excluded | 0.019261 | 0.01075 |
| PC ae C40:6 | Phosphatidylcholine acyl-alkyl C40:6 | 100.0 | 14.7 | Excluded | 0.005087 | 0.00089 |
| PC ae C42:0 | Phosphatidylcholine acyl-alkyl C42:0 | 100.0 | 3.2 | Excluded | 0.010024 | 0.0036 |
| PC ae C42:1 | Phosphatidylcholine acyl-alkyl C42:1 | 100.0 | 17.1 | Excluded | 0.005233 | 0.00442 |
| PC ae C42:2 | Phosphatidylcholine acyl-alkyl C42:2 | 100.0 | 29.3 | Excluded | 0.042102 | 0.04473 |
| PC ae C42:3 | Phosphatidylcholine acyl-alkyl C42:3 | 100.0 | 45.3 | Excluded | 0.003433 | 0.0019 |
| PC ae C42:4 | Phosphatidylcholine acyl-alkyl C42:4 | 100.0 | . | Excluded | 0.004575 | 0.00164 |
| PC ae C42:5 | Phosphatidylcholine acyl-alkyl C42:5 | 100.0 | 2.9 | Excluded | 0.013242 | 0.00464 |
| PC ae C44:3 | Phosphatidylcholine acyl-alkyl C44:3 | 100.0 | 30 | Excluded | 0.005385 | 0.0022 |
| PC ae C44:4 | Phosphatidylcholine acyl-alkyl C44:4 | 100.0 | 6.1 | Excluded | 0.083291 | 0.15242 |
| PC ae C44:5 | Phosphatidylcholine acyl-alkyl C44:5 | 100.0 | 23.0 | Excluded | 0.075088 | 0.00194 |
| PC ae C44:6 | Phosphatidylcholine acyl-alkyl C44:6 | 100.0 | 20.8 | Excluded | 0.008064 | 0.00661 |
| PC a C14:0 | lysoPhosphatidylcholine acyl C14:0 | 100.0 | 3.9 | Excluded | 0.008473 | 0.00188 |
| PC a C16:0 | lysoPhosphatidylcholine acyl C16:0 | 100.0 | 43.3 | Excluded | 0.001784 | 0.00092 |
| PC a C16:1 | lysoPhosphatidylcholine acyl C16:1 | 98.8 | 48.7 | Excluded | 0.028027 | 0.02614 |
| PC a C17:0 | lysoPhosphatidylcholine acyl C17:0 | 60.5 | 21.6 | Excluded | 0.003237 | 0.00151 |
| PC a C18:0 | lysoPhosphatidylcholine acyl C18:0 | 100.0 | 18.4 | Excluded | 0.00551 | 0.00465 |
| PC a C18:1 | lysoPhosphatidylcholine acyl C18:1 | 37.0 | 29.9 | Excluded | 0.415221 | 0.44403 |
| PC a C18:2 | lysoPhosphatidylcholine acyl C18:2 | 59.3 | 29 | Excluded | 0.047612 | 0.04795 |
| PC a C20:3 | lysoPhosphatidylcholine acyl C20:3 | 100.0 | 16.2 | Excluded | 0.00841 | 0.0046 |
| PC a C20:4 | lysoPhosphatidylcholine acyl C20:4 | 69.1 | 51.7 | Excluded | 0.006516 | 0.00467 |
| PC a C24:0 | lysoPhosphatidylcholine acyl C24:0 | 100.0 | 10.0 | Excluded | 0 | 0 |
| PC a C26:0 | lysoPhosphatidylcholine acyl C26:0 | 65.4 | 28.3 | Excluded | 0.391312 | 0.42212 |
| PC a C26:1 | lysoPhosphatidylcholine acyl C26:1 | 53.1 | 39.6 | Excluded | 0.014799 | 0.01055 |
| PC a C28:0 | lysoPhosphatidylcholine acyl C28:0 | 100.0 | 21.5 | Excluded | 0.038754 | 0.03624 |
| PC a C28:1 | lysoPhosphatidylcholine acyl C28:1 | 100.0 | 34.2 | Excluded | 0.018246 | 0.01748 |
| PC a C6:0 | lysoPhosphatidylcholine acyl C6:0 | 0.0 | 16.5 | Used | 0.019802 | 0.01883 |
| SM (OH) C14:1 | Hydroxysphingomyeline C14:1 | 100.0 | . | Excluded | 0 | 0 |
| SM (OH) C16:1 | Hydroxysphingomyeline C16:1 | 6.2 | 87.1 | Excluded | 0.004447 | 0 |
| SM (OH) C22:1 | Hydroxysphingomyeline C22:1 | 0.0 | 22.0 | Used | 0.038277 | 0.00259 |
| SM (OH) C22:2 | Hydroxysphingomyeline C22:2 | 11.1 | 68.8 | Excluded | 0.007418 | 0.00025 |
| SM (OH) C24:1 | Hydroxysphingomyeline C24:1 | 3.7 | 66.7 | Excluded | 0.007766 | 0.0008 |
| SM C16:0 | Sphingomyeline C16:0 | 0.0 | 5.8 | Used | 0.222754 | 0.0047 |
| SM C16:1 | Sphingomyeline C16:1 | 0.0 | 42.7 | Excluded | 0.014801 | 0.00187 |
| SM C18:0 | Sphingomyeline C18:0 | 0.0 | 11.2 | Used | 0.037636 | 0.00143 |
| SM C18:1 | Sphingomyeline C18:1 | 8.6 | 62.3 | Excluded | 0.005653 | 0.00081 |
| SM C20:2 | Sphingomyeline C20:2 | 98.8 | 223.6 | Excluded | 7.66E-05 | 0.0015 |
| SM C22:3 | Sphingomyeline C22:3 | 71.6 | 31.1 | Excluded | 0.003243 | 0.00202 |
| SM C24:0 | Sphingomyeline C24:0 | 0.0 | 10.4 | Used | 0.097295 | 0.00243 |
| SM C24:1 | Sphingomyeline C24:1 | 0.0 | 8.8 | Used | 0.106766 | 0.00074 |
| SM C26:0 | Sphingomyeline C26:0 | 22.2 | 44.7 | Excluded | 0.008098 | 0.00286 |
| SM C26:1 | Sphingomyeline C26:1 | 88.9 | 29.9 | Excluded | 0.004478 | 0.00206 |
| H1 | Hexose | 0.0 | 4.9 | Used | 622.8347 | 5.83709 |

**Table S2. Metabolites influenced by freeze and thaw cycles, when pair-wisely compared to the baseline reference**

Column one and two show 63 analyzed metabolites and their respective mean values at baseline. The following columns show the percentage of concentration change, the respective mean value, *P*-value and coefficient variance derived with from the 4 measurements of the sample after one, two and three freeze and thaw cycles. Significant *P*-values (*P* <0.05) are indicated in bold

| Metabolite | Baseline |  | Cycle 1 (26h) |  |  | Cycle 2 (52h) |  |  | Cycle 3 (78h) |  |
| --- | --- | --- | --- | --- | --- | --- | --- | --- | --- | --- |
|  | Mean [µM] | Mean [µM] | *P*-value | CV [%] | Mean [µM] | *P*-value | CV [%] | Mean [µM] | *P*-value | CV [%] |
| C0 | 58.368 | 60.178 | 0.50 | 7.4 | 60.337 | 0.49 | 7.4 | 65.745 | **8.7E-03** | 4.9 |
| C10 | 0.317 | 0.316 | 0.93 | 7.0 | 0.332 | 0.42 | 8.9 | 0.351 | **6.3E-03** | 3.6 |
| C10:1 | 1.044 | 1.052 | 0.82 | 5.3 | 1.103 | 0.33 | 8.6 | 1.147 | **5.5E-03** | 3.6 |
| C10:2 | 0.872 | 0.903 | 0.33 | 5.7 | 0.920 | 0.36 | 8.8 | 0.953 | **3.1E-03** | 1.7 |
| C12 | 0.274 | 0.285 | 0.39 | 7.5 | 0.290 | 0.34 | 9.0 | 0.311 | **9.0E-03** | 4.7 |
| C12:1 | 0.284 | 0.298 | 0.25 | 5.5 | 0.304 | 0.26 | 8.4 | 0.317 | **0.02** | 5.4 |
| C14 | 0.033 | 0.031 | 0.20 | 7.6 | 0.035 | 0.61 | 10.6 | 0.035 | 0.33 | 5.5 |
| C14:1 | 0.026 | 0.026 | 0.81 | 11.7 | 0.029 | **0.02** | 2.4 | 0.029 | **0.02** | 4.1 |
| C14:1-OH | 0.025 | 0.025 | 0.97 | 4.6 | 0.028 | 0.05 | 4.1 | 0.027 | 0.08 | 5.6 |
| C14:2 | 0.027 | 0.028 | 0.51 | 6.5 | 0.029 | 0.28 | 8.8 | 0.032 | **0.02** | 6.0 |
| C14:2-OH | 0.025 | 0.024 | 0.28 | 4.2 | 0.027 | 0.26 | 3.6 | 0.028 | 0.14 | 6.5 |
| C16 | 0.025 | 0.025 | 0.86 | 12.9 | 0.026 | 0.71 | 8.6 | 0.026 | 0.31 | 4.6 |
| C16-OH | 0.013 | 0.013 | 0.90 | 11.2 | 0.013 | 0.97 | 11.8 | 0.013 | 0.93 | 6.0 |
| C16:1-OH | 0.008 | 0.009 | 0.53 | 7.4 | 0.010 | **0.03** | 9.1 | 0.011 | **6.9E-03** | 5.3 |
| C16:2 | 0.012 | 0.011 | 0.05 | 4.5 | 0.013 | 0.44 | 11.3 | 0.012 | 0.90 | 10.1 |
| C18:2 | 0.007 | 0.006 | 0.56 | 14.2 | 0.007 | 0.82 | 24.8 | 0.006 | 0.30 | 19.3 |
| C2 | 13.809 | 14.252 | 0.42 | 4.9 | 14.655 | 0.19 | 5.9 | 15.404 | **0.01** | 3.2 |
| C3 | 1.003 | 1.016 | 0.55 | 2.3 | 1.081 | 0.13 | 7.1 | 1.142 | **7.7E-04** | 1.3 |
| (C3-DC)C4-OH | 1.197 | 1.166 | 0.48 | 6.4 | 1.227 | 0.55 | 6.4 | 1.312 | **0.02** | 4.5 |
| C3:OH | 0.096 | 0.097 | 0.86 | 8.0 | 0.097 | 0.76 | 3.9 | 0.105 | 0.25 | 11.0 |
| C3:1 | 0.027 | 0.028 | 0.78 | 6.5 | 0.032 | 0.23 | 18.0 | 0.027 | 0.77 | 6.2 |
| C4 | 7.086 | 7.185 | 0.58 | 3.4 | 7.893 | **0.02** | 5.2 | 8.177 | **1.4E-03** | 4.1 |
| C4:1 | 0.184 | 0.186 | 0.83 | 8.9 | 0.202 | 0.09 | 7.5 | 0.205 | **5.1E-03** | 3.3 |
| C5 | 0.186 | 0.183 | 0.63 | 5.8 | 0.189 | 0.70 | 4.6 | 0.204 | **0.04** | 4.5 |
| C5-DC (C6-OH) | 2.392 | 2.423 | 0.78 | 6.8 | 2.506 | 0.32 | 6.7 | 2.665 | **8.3E-03** | 2.7 |
| C5-M-DC | 0.786 | 0.792 | 0.90 | 8.1 | 0.792 | 0.86 | 6.2 | 0.864 | **0.03** | 3.3 |
| C5-OH(C3-DC-M) | 0.653 | 0.653 | 0.95 | 12.0 | 0.662 | 0.75 | 7.0 | 0.706 | **0.01** | 3.4 |
| C5:1 | 1.180 | 1.188 | 0.82 | 3.9 | 1.229 | 0.49 | 9.2 | 1.311 | **0.01** | 4.3 |
| C5:1-DC | 0.401 | 0.395 | 0.79 | 9.6 | 0.413 | 0.58 | 6.8 | 0.436 | 0.08 | 2.5 |
| C6(C4:1-DC) | 0.560 | 0.550 | 0.75 | 6.8 | 0.588 | 0.45 | 9.3 | 0.611 | 0.08 | 4.9 |
| C6:1 | 0.160 | 0.158 | 0.83 | 9.8 | 0.165 | 0.54 | 8.3 | 0.170 | 0.21 | 8.0 |
| C7-DC | 0.476 | 0.489 | 0.59 | 7.2 | 0.522 | 0.05 | 4.6 | 0.554 | **0.01** | 5.8 |
| C8 | 0.556 | 0.580 | 0.35 | 4.7 | 0.608 | 0.12 | 6.6 | 0.666 | **8.2E-03** | 6.8 |
| C8:1 | 2.449 | 2.569 | 0.33 | 6.8 | 2.704 | 0.05 | 5.8 | 2.972 | **2.3E-03** | 5.8 |
| C9 | 2.433 | 2.447 | 0.88 | 5.7 | 2.547 | 0.48 | 9.8 | 2.684 | **0.02** | 3.6 |
| Arg | 17.403 | 15.139 | 0.30 | 24.0 | 17.744 | 0.76 | 8.6 | 19.824 | 0.30 | 17.8 |
| Gln | 350.204 | 314.975 | 0.32 | 19.8 | 347.082 | 0.83 | 5.2 | 370.424 | 0.35 | 8.9 |
| Gly | 1631.404 | 1454.320 | 0.31 | 21.2 | 1652.026 | 0.81 | 7.5 | 1780.847 | 0.23 | 10.4 |
| His | 332.421 | 303.876 | 0.32 | 15.6 | 342.202 | 0.54 | 6.1 | 365.761 | 0.22 | 11.6 |
| Creatinin | 9405.451 | 9616.287 | 0.60 | 6.0 | 10380.952 | 0.13 | 8.8 | 10718.127 | **3.4E-03** | 2.4 |
| Met | 12.654 | 11.024 | 0.22 | 17.9 | 12.573 | 0.95 | 8.5 | 13.504 | 0.42 | 11.6 |
| Phe | 26.274 | 24.742 | 0.46 | 14.0 | 27.586 | 0.36 | 6.6 | 29.026 | 0.16 | 9.8 |
| Pro | 21.928 | 22.124 | 0.93 | 12.3 | 23.704 | 0.18 | 7.8 | 25.272 | **0.03** | 7.7 |
| Ser | 248.628 | 212.873 | 0.20 | 19.7 | 240.629 | 0.58 | 8.6 | 258.976 | 0.50 | 8.7 |
| Thr | 82.355 | 70.381 | 0.20 | 20.0 | 80.810 | 0.77 | 6.5 | 85.934 | 0.52 | 9.1 |
| Trp | 65.921 | 59.506 | 0.34 | 19.4 | 66.985 | 0.73 | 5.8 | 71.297 | 0.28 | 10.6 |
| Tyr | 72.302 | 64.198 | 0.27 | 18.9 | 75.411 | 0.49 | 8.5 | 81.299 | 0.11 | 9.9 |
| Val | 30.856 | 26.900 | 0.34 | 28.1 | 30.980 | 0.89 | 4.9 | 32.915 | 0.53 | 15.4 |
| xLeu | 50.391 | 43.776 | 0.33 | 25.5 | 51.686 | 0.72 | 10.5 | 53.680 | 0.36 | 9.8 |
| Lyso PC a C6:0 | 0.073 | 0.057 | 0.17 | 30.1 | 0.063 | 0.32 | 27.2 | 0.072 | 0.89 | 12.2 |
| PC aa C28:1 | 0.080 | 0.076 | 0.26 | 6.2 | 0.083 | 0.51 | 7.3 | 0.078 | 0.70 | 9.6 |
| PC aa C32:0 | 0.018 | 0.019 | 0.54 | 11.0 | 0.020 | 0.18 | 6.7 | 0.021 | 0.07 | 4.6 |
| PC aa C34:1 | 0.084 | 0.079 | 0.65 | 8.0 | 0.088 | 0.59 | 10.2 | 0.083 | 0.95 | 14.4 |
| PC aa C34:4 | 0.008 | 0.008 | 0.62 | 29.8 | 0.008 | 0.73 | 26.2 | 0.009 | 0.88 | 30.6 |
| PC aa C36:1 | 0.042 | 0.040 | 0.76 | 9.5 | 0.046 | 0.41 | 14.0 | 0.044 | 0.73 | 14.1 |
| PC ae C34:1 | 0.014 | 0.018 | **0.04** | 14.5 | 0.018 | **0.01** | 10.4 | 0.017 | 0.05 | 14.2 |
| PC ae C38:6 | 0.075 | 0.077 | 0.71 | 7.7 | 0.075 | 0.93 | 6.2 | 0.072 | 0.58 | 5.7 |
| SM (OH) C22:1 | 0.038 | 0.043 | 0.33 | 14.8 | 0.042 | 0.43 | 14.4 | 0.051 | **0.04** | 13.9 |
| SM C16:0 | 0.223 | 0.215 | 0.35 | 4.0 | 0.231 | 0.51 | 8.8 | 0.255 | **5.0E-03** | 4.6 |
| SM C18:0 | 0.038 | 0.029 | 0.06 | 19.7 | 0.033 | 0.23 | 15.3 | 0.040 | 0.57 | 14.3 |
| SM C24:0 | 0.097 | 0.099 | 0.88 | 16.1 | 0.099 | 0.79 | 7.8 | 0.102 | 0.42 | 7.2 |
| SM C24:1 | 0.107 | 0.103 | 0.70 | 17.0 | 0.101 | 0.56 | 19.9 | 0.119 | 0.12 | 9.0 |
| H1 | 622.835 | 652.282 | 0.24 | 5.6 | 698.853 | 0.07 | 8.3 | 748.958 | **3.1E-04** | 3.4 |
